# Inter‐rater Reliability of the Glasgow Coma Scale Between Triage and Resuscitation Nurses After Standardized Training

**DOI:** 10.1155/jonm/5539097

**Published:** 2025-12-15

**Authors:** Yangchun Zhang, Cairong Liu, Feng Chen, Na Ma, Yun Cao, Xufeng Chen, Xueli Ji

**Affiliations:** ^1^ Emergency Department, The First Affiliated Hospital With Nanjing Medical University, Nanjing, Jiangsu, China, njmu.edu.cn

**Keywords:** emergency nursing, Glasgow Coma Scale, inter-rater reliability, standardized assessment, trauma triage

## Abstract

**Aims:**

To evaluate Glasgow Coma Scale (GCS) inter‐rater reliability between triage and resuscitation nurses following standardized training and identify factors influencing scoring consistency in a high‐volume Chinese trauma center.

**Design:**

A prospective observational study adhering to STROBE guidelines.

**Methods:**

Data were collected from 875 trauma patients and 71 nurses (30 triage and 41 resuscitation) between April and December 2024. Nurses completed a 19‐h standardized GCS training program. Patients underwent two independent GCS assessments within ≤ 5 min: first by triage nurses and then by resuscitation nurses. Inter‐rater reliability was assessed using percentage agreement, weighted Cohen’s kappa, intraclass correlation coefficients (ICC), and correlation analyses. Multivariate logistic regression identified factors associated with scoring consistency.

**Results:**

GCS components and total scores demonstrated high inter‐rater reliability (weighted kappa: 0.85–0.96; ICC: 0.85–0.96). Perfect agreement occurred in 65.3% of total GCS scores, with 89.8% differing by ≤ 1 point. Verbal response showed the highest component agreement (87.1%), followed by eye opening (86.5%) and motor response (75.1%). Patient characteristics significantly influenced consistency, with traumatic brain injury (adjusted OR = 0.22, *p* < 0.001) and injury severity scores ≥ 16 (adjusted OR = 0.34, *p* < 0.001) associated with reduced agreement. Nurses′ experience, education, and professional rank did not significantly affect reliability.

**Conclusion:**

Poststandardized training, GCS assessments showed high inter‐rater reliability among emergency nurses, though motor response demonstrated comparatively lower consistency. Injury severity and traumatic brain injury were primary factors affecting scoring agreement, indicating the need for heightened vigilance when assessing critically ill patients.

**Relevance to Clinical Practice:**

Within China’s developing prehospital triage infrastructure, standardized GCS training can substantially improve neurological assessment reliability in emergency settings, facilitating accurate identification of critically injured patients and optimizing resource allocation.

**Patient or Public Contribution:**

No patient or public contribution.

## 1. Introduction

Trauma remains a leading cause of morbidity and mortality worldwide, necessitating prompt and precise neurological assessment to guide early intervention [1]. The Glasgow Coma Scale (GCS) stands as the most widely adopted tool for evaluating consciousness in trauma patients [2]. This scale serves as a critical component of trauma triage, enabling emergency nurses to quickly identify severely injured patients, determine when to activate trauma teams, and predict clinical outcomes [3–5].

In China, unlike many developed countries, prehospital triage and graded trauma care systems remain underdeveloped, causing an influx of minor‐to‐moderate trauma cases at provincial trauma centers [6]. This overcrowding strains emergency resources and hampers nurses′ ability to promptly identify severely injured patients. In the absence of effective prehospital trauma triage, nurses must depend on in‐hospital tools such as the GCS for rapid, accurate assessments, highlighting the critical need for scoring reliability. Inconsistencies in GCS scoring among emergency nurses can result in variable triage decisions, intervention delays, and disparate treatment plans.

This study evaluates the inter‐rater reliability of standardized GCS assessment among emergency nurses in a high‐volume Chinese trauma center. The findings will inform improvements in trauma triage strategies and highlight the necessity of standardized GCS assessment protocols in emergency nursing practice.

## 2. Background

Emergency nurses serve a critical role in early trauma assessment and triage, requiring swift and precise evaluation of neurological status. The GCS represents a fundamental clinical indicator for assessing traumatic brain injury severity and overall trauma classification [3–5]. This tool effectively stratifies patients according to injury severity [7], informing crucial early clinical decisions, including intubation [8], ICU admission [9], and trauma team activation [1]. Additionally, GCS scores contribute to predicting both immediate and long‐term patient outcomes [10].

Despite its widespread adoption, GCS scoring reliability remains problematic, especially in high‐pressure emergency environments. Research has documented moderate to poor inter‐rater reliability for GCS assessments [8, 11–13]. These scoring inconsistencies stem from multiple factors: treatment interventions (particularly sedation and intubation) that interfere with neurological assessment, variations in patient consciousness levels, differences in observer training and experience, inconsistent stimulation techniques, and environmental factors [14, 15]. Within China’s high‐volume trauma centers, these challenges intensify, elevating misclassification risks and potentially compromising patient outcomes.

Standardized training programs for emergency nurses have been proposed to address GCS scoring variability [15–17]. Effective interventions incorporate structured assessment protocols [14], scenario‐based learning approaches [8], and systematic reinforcement of scoring criteria [18]. These educational strategies may enhance both GCS scoring reliability and subsequent clinical decision‐making quality. However, research examining the effectiveness of standardized GCS training in authentic clinical environments remains limited, particularly regarding its impact on inter‐rater reliability.

Knowledge gaps in this area necessitate a prospective observational study to evaluate inter‐rater reliability of GCS assessments among emergency nurses following standardized protocols. This research will provide essential insights into GCS reliability and clinical utility, reinforcing its position as an evidence‐based neurological assessment tool in emergency nursing practice.

## 3. Methods

### 3.1. Study Design

This prospective observational study evaluated the inter‐rater reliability of standardized GCS assessment practices. Researchers collected data from patients admitted to a Chinese provincial trauma center between April and December 2024. The investigation employed no experimental interventions or randomization procedures. Methodological design and reporting adhered to the Strengthened Reporting of Observational Studies in Epidemiology (STROBE) guidelines.

### 3.2. Study Setting

This study was conducted at the first provincial trauma center in Jiangsu Province, China, which was established in December 2017 within a tertiary general hospital. The center’s mission is to enhance trauma care efficiency, improve care quality, and reduce mortality and disability rates among trauma patients. As a leading trauma care facility in the province, findings from this study may have significant implications for improving trauma triage protocols and neurological assessment procedures across high‐volume trauma centers throughout China.

### 3.3. Participant Selection

#### 3.3.1. Nurses

Emergency department registered nurses were eligible for participation if they: (1) worked in both triage and resuscitation areas; (2) completed the standardized GCS training program successfully; and (3) possessed a minimum 1 year of emergency nursing experience. Exclusion criteria included failure of GCS training assessment, noninvolvement in both triage and resuscitation during the study period, or transfer from the emergency department before study completion.

#### 3.3.2. Patients

Adult patients (≥ 18 years) admitted to the Emergency Resuscitation Unit (ERU) meeting any trauma team activation criteria [1] were included if they underwent GCS assessment at both triage and resuscitation with an inter‐assessment interval of ≤ 5 min. To minimize confounding factors affecting GCS reliability, exclusion criteria comprised (1) use of sedatives, neuromuscular blocking agents, alcohol, or addictive substances within 24 h preassessment; (2) severe periorbital edema or damage to eyeballs, optic nerves, or motor nerves; (3) pre‐existing speech, hearing, or limb movement disorders; (4) dementia or psychiatric disorders; (5) tracheal intubation prior to initial GCS evaluation; or (6) incomplete assessment data.

### 3.4. GCS Assessment Training

Before study initiation, all participating emergency nurses completed a comprehensive, standardized GCS training program based on the official GCS educational framework (https://www.glasgowcomascale.org). The structured 19‐h protocol comprised five sequential components: (1) a 4‐h theoretical foundation covering neurological assessment principles, GCS development and validation, component‐specific techniques, and common scoring pitfalls; (2) a 2‐h observational learning module with expert‐narrated videos demonstrating standardized procedures and visual guides for response differentiation; (3) a 1‐h knowledge validation via a 50‐item multiple‐choice examination requiring ≥ 90% passing score; (4) an 8‐h practical competency development incorporating simulation with standardized patients, high‐fidelity mannequin scenarios, paired assessment exercises, and video‐recorded performance debriefing; and (5) a 4‐h clinical integration with supervised practice and paired assessments with experienced evaluators.

All materials underwent rigorous translation and cultural adaptation into Chinese following established protocols to ensure conceptual equivalence. A multidisciplinary team developed and oversaw the project, consisting of a neurologist and emergency medicine specialist (both Associate Medical Directors with > 10 years′ experience) and an advanced practice emergency nurse (with > 15 years′ acute care experience). Competency verification required: ≥ 90% accuracy on theoretical assessments, successful completion of all simulation scenarios, inter‐rater reliability with *κ* ≥ 0.90 in paired assessments, and passing a comprehensive final evaluation. Only nurses meeting all requirements received GCS assessment certification. Quarterly refresher training and competency verification maintained assessment quality throughout the study period, with all participating nurses required to demonstrate continued adherence to the same certification standards throughout the study duration.

### 3.5. Clinical GCS Assessment Protocol

#### 3.5.1. Assessment Procedure

Triage nurses performed initial GCS assessment upon patient arrival based on predefined trauma team activation criteria. Resuscitation nurses subsequently conducted a second independent GCS evaluation following patient transfer to the resuscitation room. Assessment interval was maintained at ≤ 5 min to minimize effects of potential condition changes. Both assessments were independently documented in separate emergency information system modules—triage and resuscitation—ensuring blinded evaluation. Both utilized standardized structured forms where nurses selected appropriate scores via mouse click, with automatic recording of component scores and total score calculation. Each independent assessment was completed within 1 min.

**Figure 1:**
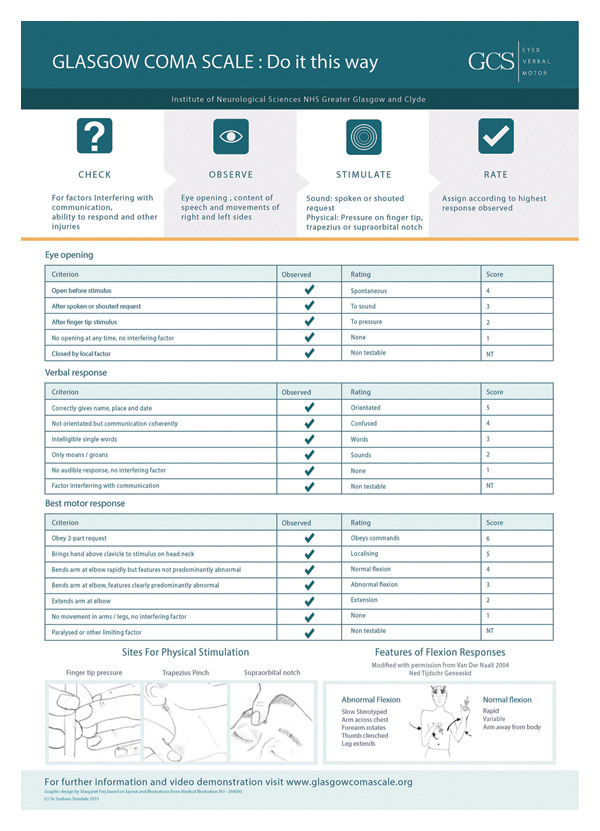
GCS assessment aid.

The GCS assessment protocol follows four steps recommended by the GCS official guidelines (Figure 1).

1. Check: Before assessment, nurses identified factors potentially influencing patient response: language/cultural barriers, intellectual/sensory deficits, pathological states (intubation and tracheostomy), medication effects (sedatives and muscle relaxants), and structural damage affecting assessment (skull fractures, aphasia, and spinal injuries).
2. Observe: Nurses evaluated three key components: eye opening, verbal response, and motor response.
3. Stimulate: A standardized protocol progressed from verbal stimuli (speaking/calling) to physical stimuli (fingertip pressure, trapezius squeeze, and supraorbital pressure) with increasing intensity for a maximum 10 s.
4. Score: The highest observed response in each category (eye opening, verbal response, motor response [EVM]) was recorded.

### 3.6. Component‐Specific Assessment Protocols

#### 3.6.1. Eye‐Opening Assessment

Eye opening was evaluated on a four‐point scale. For spontaneously open eyes, “Spontaneous” was recorded. Otherwise, verbal stimulation was applied by introducing oneself and requesting eye opening, increasing volume if needed. Eye opening in response was recorded as “To Sound.” If no response occurred, peripheral physical stimulation was applied, starting with fingernail pressure and increasing in intensity up to 10 s until response. Eye opening to physical stimuli was recorded as “To Pressure”; absence as “None.” When local factors prevented assessment (e.g., periorbital edema), “Untestable” was documented.

#### 3.6.2. Verbal Response Assessment

Verbal response was evaluated on a five‐point scale. Patients were asked their name, location, and current month. Correct responses were scored as “Oriented.” Incorrect orientation with phrases/sentences was recorded as “Confused.” Single‐word responses were documented as “Words,” while incomprehensible sounds were documented or moaning as “Sounds.” No vocalization was recorded as “None.” For patients with conditions preventing verbal assessment (intubation, aphasia), “Untestable” was documented.

#### 3.6.3. Motor Response Assessment

Motor response was evaluated on a six‐point scale. Patients were asked to perform two‐step actions (grasp/release examiner’s fingers or open mouth/extend tongue). Successful performance was scored as “Obeys Commands.” For upper extremity immobility due to spinal injury, alternative oral commands were used.

For noncompliant patients, painful stimuli were applied in a standardized sequence. Peripheral painful stimulus (fingernail pressure) was applied first. If no response was observed, central painful stimuli were then applied, beginning with a trapezius squeeze, increasing pressure for up to 10 s until the best response was observed. If unresponsive, supraorbital pressure was applied by positioning the thumb at the supraorbital notch and increasing pressure for up to 10 s.

Hand movement above the clavicle was recorded as “Localizes.” Arm flexion below the clavicle was classified as normal or abnormal. Normal flexion (“Withdraws”) featured rapid elbow flexion away from stimulus. Abnormal flexion involved slow elbow flexion across the body (“Flexion”). Elbow extension was documented as “Extension.” No response was recorded as “None.” For assessment‐preventing factors (e.g., pharmacological paralysis), “Untestable” was documented. With asymmetrical limb responses, the better response was recorded.

### 3.7. Mnemonic Aids for Clinical Application

Clinical staff used standardized mnemonics for accurate GCS scoring.

• Eye opening: “ESPN” (Eye Opening Spontaneously, To Sound, To Pain, No Response)
• Verbal response: “Our Country WIN” (Oriented, Confused, Words, Incomprehensible Sounds, No Response)
• Motor response: “Can’t Live Without FANs” (Obeys Commands, Localizes to Pain, Withdraws to Pain, Flexion, Abnormal Extension, No Response)

### 3.8. Data Collection

Data were collected using standardized spreadsheets from the hospital’s Management Information System and Emergency Patient Care Information System. Variables included.

1. Nurse characteristics: age, education level, emergency nursing experience years, professional grade
2. Patient information: age, gender, assessment time (daytime: 07:00–19:00; night: 19:00–07:00), arrival mode (ambulance/nonambulance), traumatic brain injury presence (yes/no)
3. Assessment parameters: GCS component scores (eye, verbal, and motor) for triage and resuscitation assessments, total GCS scores, and injury severity scores (ISSs) based on Abbreviated Injury Scale calculated via the hospital trauma system

Research assistants followed standardized extraction protocols. For quality assurance, 10% of records underwent independent double extraction with discrepancies resolved by study team consensus.

### 3.9. Statistical Analysis

Descriptive statistics included frequencies/percentages for categorical variables and means with ranges or medians with interquartile ranges for numerical variables based on distribution. The Kolmogorov–Smirnov test assessed normality. Inter‐rater reliability between GCS assessments used multiple approaches.

1. Agreement measures: percentage of identical scores, weighted Cohen’s kappa (κw, 95% CI) with quadratic weighting (≤ 0.40 poor, 0.41–0.60 moderate, 0.61–0.80 substantial, > 0.80 almost perfect), and intraclass correlation coefficient (ICC) using two‐way random effects model (< 0.50 poor, 0.50–0.75 moderate, 0.75–0.90 good, > 0.90 excellent).
2. Correlation analysis: Kendall’s *τ*‐b coefficient, Spearman’s rank correlation (*ρ*, 95% CI), and squared Spearman’s coefficient (*ρ* ^2^) for variance explanation.
3. Visualization: histograms showing score differences and Bland–Altman plots evaluating mean differences and agreement limits (mean ± 1.96SD).

Multivariate logistic regression identified factors associated with GCS consistency (binary outcome: consistent vs. inconsistent scoring). Independent variables included nurse factors (age, experience, education, and rank) and patient factors (sex, age, arrival mode, assessment timing, traumatic brain injury, injury severity [ISS ≥ 16 or < 16]). Adjusted odds ratios (95% CI) were calculated.

All analyses were conducted using MedCalc Software (Version 20.010, https://www.medcalc.org), R Statistical Software (Version 4.2.2, http://www.R-project.org, The R Foundation), and the Free Statistics analysis platform (Version 2.1.1, Beijing, China, http://www.clinicalscientists.cn/freestatistics). A two‐sided *p* value < 0.05 was considered statistically significant.

### 3.10. Ethical Considerations

This study was approved by the Ethics Committee of the First Affiliated Hospital with Nanjing Medical University (Approval Number: 2025‐SR‐164). Since GCS assessment is part of routine emergency care, the requirement for patient informed consent was waived. All data were de‐identified before analysis to ensure compliance with ethical guidelines and data protection regulations.

## 4. Results

### 4.1. Participant Characteristics

Table 1 summarizes demographic and clinical characteristics of study participants.

**Table 1:** Characteristics of patients and nurses.

Characteristic	No. (%)
Total number of patients	**875**
Age, *y*, mean (range)	55.6 (18–100)
Sex	
Male	612
Female	263
Mode of arrival	
Ambulance	81 (9.3)
Nonambulance	794 (90.7)
Consultation time	
Daytime (07:00–19:00)	520 (59.4)
Evenings and nights (19:00–07:00)	355 (40.6)
Brain injury	
Yes	231 (26.4)
No	644 (74.9)
ISS ≥ 16	
Yes	220 (25.1)
No	655 (74.9)
Total number of triage nurses	**30**
Age, *y*, mean (range)	35.6 (30–45)
Experience of working in emergency, *y*, mean (range)	14.23 (9–25)
Professional level	
N4	1 (3.3)
N3	18 (60.0)
N2	11 (36.7)
Academic qualifications	
Master	1 (3.3)
Bachelor	29 (96.7)
Total number of resuscitation nurses	**41**
Age, *y*, mean (range)	28.39 (23–33)
Experience of working in emergency, *y*, mean (range)	6.31 (2–9)
Professional level	
N3	3 (9.8)
N2	34 (82.9)
N1	4 (9.8)
Academic qualifications	
Master	1 (2.4)
Bachelor	37 (90.2)
Associate	3 (7.3)

*Note:* The bold values represent the key total figures (patients and nurses) for quick identification of core statistical data.

Nurse characteristics: 30 triage nurses (mean age 35.6 years [30–45], mean emergency experience 14.23 years [9–24], 96.7% bachelor’s degree) and 41 resuscitation nurses (mean age 28.39 years [23–33], mean emergency experience 6.31 years [2–9], 90.2% bachelor’s degree, 82.9% N2 professional level).

Patient characteristics (*n* = 875): mean age 55.6 years [18–100], 70.0% male (612/875), 9.3% ambulance arrivals (81/875), 59.4% daytime presentations (520/875), 26.4% traumatic brain injuries (231/875), and 25.1% severe trauma with ISS ≥ 16 (220/875). Of 1036 patients meeting trauma team criteria, 875 were included per study criteria.

### 4.2. Inter‐rater Reliability of GCS Scoring

Table 2 shows high inter‐rater agreement across all GCS components: eye opening (κw = 0.87, 95% CI: 0.83–0.90), motor (κw = 0.85, 95% CI: 0.83–0.88), and verbal (κw = 0.93, 95% CI: 0.92–0.95), with the highest agreement for total score (κw = 0.96, 95% CI: 0.95–0.96). ICC analyses confirmed good reliability (range: 0.85–0.96).

**Table 2:** Measures of inter‐rater reliability between paired ratings.

Measure	Eye	Verbal	Motor	Total
Agreement, %	86.5	87.1	75.1	65.3
Kendall’s *τ*‐b	0.81 (0.78–0.85)	0.89 (0.86–0.91)	0.77 (0.73–0.80)	0.82 (0.79–0.84)
Spearman’s (*ρ*) (95% CIs)	0.84 (0.82–0.86)	0.92 (0.91–0.93)	0.82 (0.79–084)	0.87 (0.85–0.89)
Spearman’s (*ρ* ^2^), %	70.0	83.9	66.4	75.9
Weighted Cohen’s kappa	0.87 ± 0.02	0.93 ± 0.01	0.85 ± 0.01	0.96 ± 0.01
ICC	0.87 (0.84–0.88)	0.93 (0.92–0.94)	0.85 (0.83–0.87)	0.96 (0.95–0.98)

*Note:* Weighted Cohen’s kappa interpretation: ≤ 0.40 poor, 0.41–0.60 moderate, 0.61–0.80 substantial, > 0.80 almost perfect agreement. ICC interpretation: < 0.50 poor, 0.50–0.75 moderate, 0.75–0.90 good, > 0.90 excellent reliability.

Strong correlations existed between triage and resuscitation assessments (Spearman *ρ*: 0.82–0.92). Squared correlation coefficients showed 66.4%–83.9% of variance in one assessment explained by another. Verbal response showed highest consistency (*ρ* ^2^ = 83.9%), while motor response showed lowest (*ρ* ^2^ = 66.4%).

### 4.3. Distribution of Rating Differences

Paired score differences appear in Figure 2 (histogram) and Figure 3 (Bland–Altman plot). Table 3 shows 65.3% (571/875) of GCS total scores were identical, 89.8% (785/875) differed by < 1 point, and 97.3% (851/875) by < 2 points. Component perfect agreement percentages were verbal (87.1%), eye opening (86.5%), and motor (75.1%). Discrepancies < 1 point: verbal (97.4%), eye opening (97.7%), and motor (94.9%).

Figure 2Histograms of the difference between the paired ratings. a, Eye component; b, verbal component; c, motor component; d, total.

**Figure:**
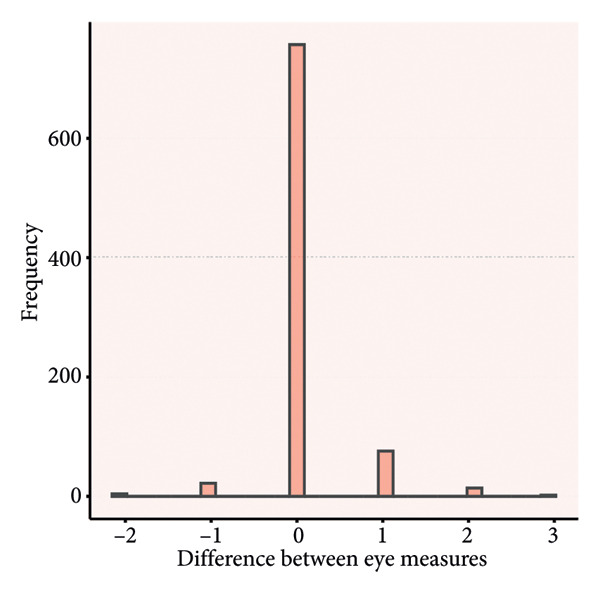
(a)

**Figure:**
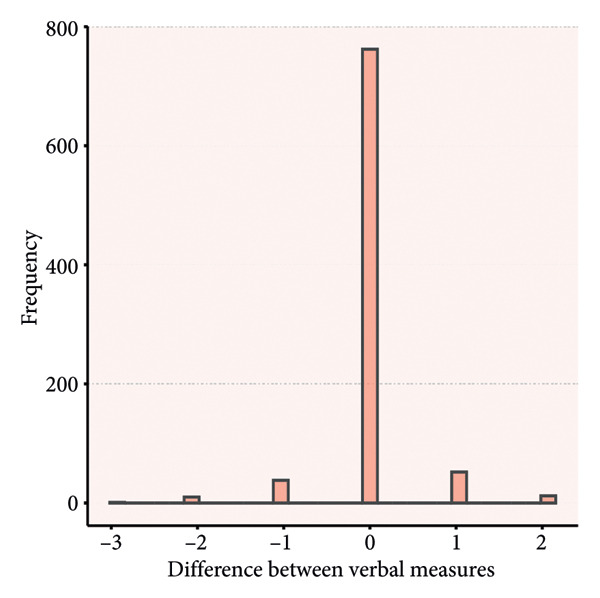
(b)

**Figure:**
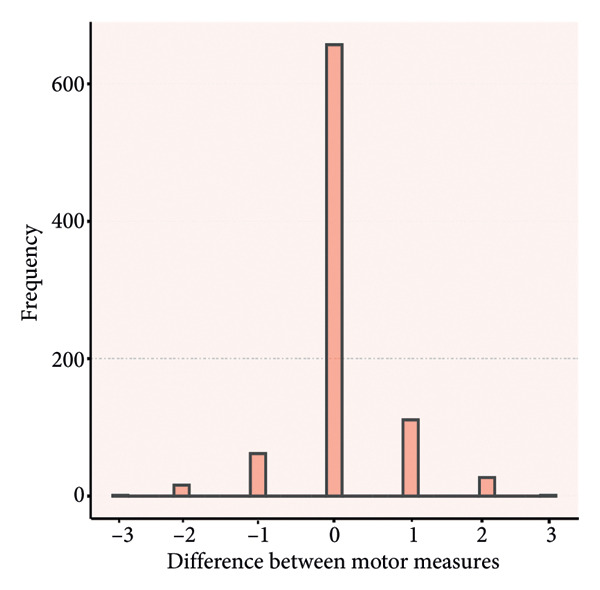
(c)

**Figure:**
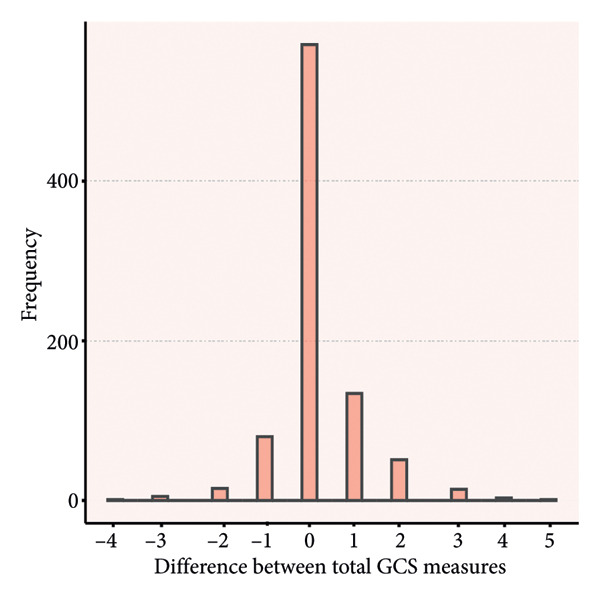
(d)

**Table 3:** Absolute differences between paired ratings.

Absolute difference	Eye (%)	Verbal (%)	Motor (%)	Total (%)
0	757 (86.5)	762 (87.1)	657 (75.1)	571 (65.3)
1	98 (11.2)	90 (10.3)	173 (19.8)	214 (24.5)
2	18 (2.1)	22 (2.5)	43 (4.9)	66 (7.5)
3	2 (0.2)	1 (0.1)	2 (0.2)	19 (2.2)
4	0	0	0	4 (0.4)
5	0	0	0	1 (0.1)

Bland–Altman analyses (Figure 3) showed evenly distributed differences around zero, indicating no systematic bias. Limits of agreement showed differences within: eye response (±0.43 points), verbal response (±0.39 points), motor response (±0.75 points), and total score (±1.08 points), confirming highest motor response variability and best verbal response agreement.

Figure 3Bland–Altman plots of absolute differences versus means. a, Eye component; b, verbal component; c, motor component; d, total.

**Figure:**
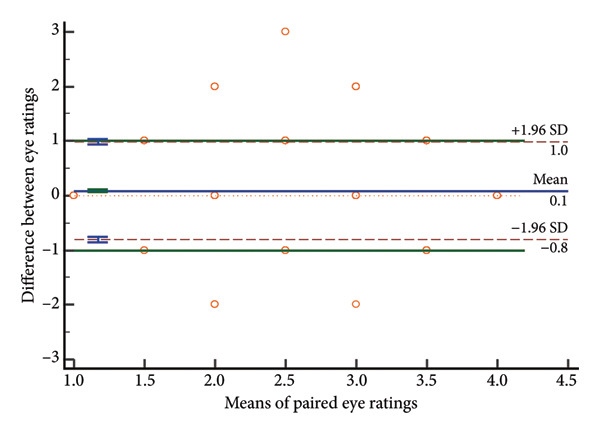
(a)

**Figure:**
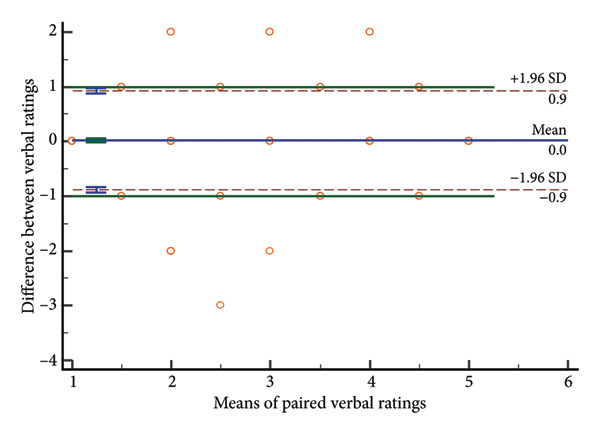
(b)

**Figure:**
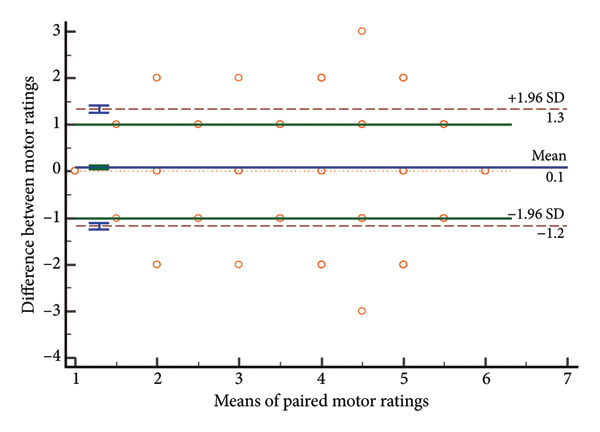
(c)

**Figure:**
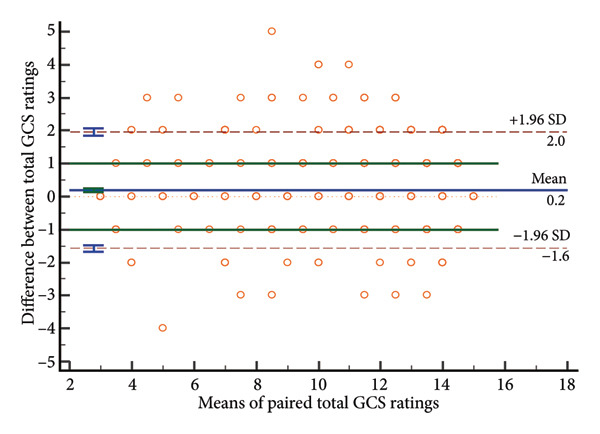
(d)

### 4.4. Factors Influencing GCS Scoring Consistency

Multivariate logistic regression (Table 4) showed no significant correlation between GCS score consistency and nurse factors (education, experience, and rank) or patient demographics (gender, age, transport mode, and visit time) (*p* > 0.05). Clinical characteristics significantly affected concordance: Patients with ISS ≥ 16 showed lower GCS assessment concordance than those with ISS < 16 (adjusted OR = 0.34, 95% CI: 0.24–0.47, *p* < 0.001). Similarly, patients with traumatic brain injury showed lower concordance than those without (adjusted OR = 0.22, 95% CI: 0.16–0.31, *p* < 0.001). Results suggest injury severity and type significantly influence GCS score consistency, while assessor characteristics and assessment timing have minimal impact.

**Table 4:** Results of multivariate logistic regression analyses of the consistency of GCS total scores.

Variable	crude.OR (95%CI)	crude.*p* value	adj.OR (95%CI)	adj.*p* value
*Patients*				
Sex				
Male	1 (ref)		1 (ref)	
Female	0.80 (0.59–1.07)	0.136	0.80 (0.58–1.12)	0.191
Age, years	0.99 (0.99–1.00)	0.155	1.00 (0.99–1.01)	0.812
Mode of arrival				
Nonambulance	1 (ref)		1 (ref)	
Ambulance	0.59 (0.35–1.00)	0.048	0.78 (0.44–1.39)	0.405
Consultation time				
Daytime (07:00–19:00)	1 (ref)		1 (ref)	
Evenings and nights (19:00–07:00)	1.27 (0.95–1.69)	0.101	1.18 (0.86–1.62)	0.306
ISS ≥ 16				
No	1 (ref)		1 (ref)	
Yes	0.33 (0.24–0.45)	**< 0.001**	0.34 (0.24–0.47)	**< 0.001**
Brain injury				
No	1 (ref)		1 (ref)	
Yes	0.22 (0.16–0.30)	**< 0.001**	0.22 (0.16–0.31)	**< 0.001**

*Triage nurses*				
Age, years	0.99 (0.96–1.03)	0.732	1.02 (0.88–1.18)	0.780
Experience, years	0.99 (0.96–1.02)	0.531	0.97 (0.85–1.1)	0.626
Professional level				
N2	1 (ref)		1 (ref)	
N3	0.96 (0.72–1.30)	0.807	1.11 (0.70–1.76)	0.666
N4	1.21 (0.49–2.99)	0.682	1.46 (0.50–4.30)	0.492
Academic qualifications				
Master	1 (ref)		1 (ref)	
Bachelor	1.22 (0.50–3.01)	0.661	1.46 (0.50–4.30)	0.492

*Resuscitation nurses*				
Age, years	0.97 (0.91–1.04)	0.408	0.98 (0.84–1.14)	0.791
Experience, years	0.97 (0.89–1.05)	0.431	0.99 (0.82–1.20)	0.952
Professional level				
N1	1 (ref)		1 (ref)	
N2	0.99 (0.59–1.66)	0.967	1.58 (0.54–4.65)	0.405
N3	0.84 (0.41–1.76)	0.652	1.58 (0.36–6.99)	0.546
Academic qualifications				
Associate	1 (ref)		1 (ref)	
Master	0.91 (0.49–1.68)	0.756	0.72 (0.22–2.34)	0.580
Bachelor	0.85 (0.30–2.43)	0.760	0.58 (0.10–3.54)	0.556

*Note:* The bold values represent highly statistically significant *p* values, indicating strong evidence of real associations between “ISS ≥ 16,” “Brain injury,” and the outcome variable (not due to chance).

## 5. Discussion

This study evaluated GCS inter‐rater reliability among emergency nurses after standardized training and identified factors affecting score consistency. In China’s limited prehospital triage system, accurate neurological assessment by emergency nurses critically influences triage decisions, trauma team activation, and clinical outcomes. Results demonstrated high overall reliability after training (κw: 0.85–0.96; ICC: 0.85–0.96), substantially exceeding the “moderate to poor” reliability characterized by Reith et al.′s systematic review [14], where only 13% of studies achieved methodologically sound results. Our perfect agreement rate of 65.3% for total GCS scores and ICC values ranging from 0.85 to 0.96 markedly surpasses those typically reported in emergency settings, such as Gill et al.′s kappa values of 0.40–0.72 [12]. However, 34.7% of total GCS assessments showed differences despite short intervals (≤ 5 min), with motor response showing the lowest concordance (75.1%). Patient clinical factors (ISS scores, traumatic brain injury) significantly affected GCS consistency, while nurse qualifications (experience and education) had no significant impact. These findings provide empirical evidence for addressing acute care GCS assessment challenges and improving trauma grading strategies.

Standardized training significantly improved GCS component reliability, though motor scores and total scores showed lower agreement. Complete agreement occurred in 65.3% of total GCS assessments, with 89.8% differing by < 1 point and 97.3% by < 2 points. These results exceed those in Reith et al.′s systematic review [14], which found only 13% of GCS reliability studies were methodologically sound, with significant reliability variation. Our superior concordance likely stems from rigorous methodology, including brief assessment intervals and standardized training protocols.

This finding is particularly relevant in China’s healthcare system, where limited prehospital triage results in provincial trauma centers receiving patients of all severities. Emergency nurses must rely on in‐hospital tools like GCS for rapid critical injury identification and triage decisions. Our study demonstrates that with standardized training, GCS provides reliable assessment to address resource distribution inequalities and overcrowding issues.

The GCS total score, as a simple sum of eye, verbal, and motor responses, may obscure critical clinical information. Healey et al. [20] demonstrated nearly twofold mortality rate variations among patients with identical total scores but different component combinations. For instance, a patient with “localized pain” motor response may have the same total score as one with “abnormal flexion,” despite substantial differences in neurological status and prognosis. This “numerical equivalence” masks clinical presentation heterogeneity, potentially misleading clinical decisions and trauma team activation.

Independent subscale assessment provides detailed neurological function insights: ocular responses showed high concordance (86.5%), suitable for rapid arousal screening; verbal responses also demonstrated high concordance (87.1%), offering cognitive function indicators despite endotracheal intubation scoring issues; motor responses, the strongest prognosis predictor [7, 21] essential for therapeutic strategy guidance (e.g., intracranial pressure management), showed lower agreement (75.1%), indicating need for standardized stimulation approaches.

Surveys indicate many clinicians find GCS too complex and difficult to memorize [22]. Research demonstrates GCS accuracy and assessment speed improve with scoring aids [8]. However, 33% of clinicians report not using reference cards, possibly due to emergency time constraints or the perception that using “basic tools” indicates clinical incompetence. This challenge is particularly acute in high‐intensity emergency settings, where standardized training programs are essential [18, 22, 23]. Our study implemented mnemonics for GCS score memorization and an electronic point‐and‐click recording system, eliminating calculation errors while improving assessment accuracy and reliability. This approach demonstrated high inter‐rater agreement (total score ICC = 0.96).

Standardized GCS assessment significantly improved inter‐assessor agreement, particularly for pain stimuli application. Findings align with Cook and Braine [24], who identified pain stimulus methodology as the primary GCS application controversy. Their study revealed significant variations in stimulation location, duration, and intensity directly affecting assessment reliability. Our standardized protocol defines precise stimulus parameters (location, intensity, and duration ≤ 10 s), aligning with established physiological response times in the literature. With pain signaling velocities of 2–40 m/s, responses occur almost instantaneously, eliminating the need for extended stimulation [24]. By standardizing application parameters, we have minimized the assessment practice variations documented by Ayda [23] and Reith et al. [14], who identified significant inter‐assessor differences in stimulation methodology. Notably, our protocol implements a structured stimulation sequence (progressing from fingertip pressure to trapezius squeeze or supraorbital pressure), conforming to official GCS recommendations. This systematic approach eliminates the assessment bias from inconsistent stimulation methods identified by Singh et al. [25]. These standardization measures enhanced inter‐assessor reliability while reducing the risk of stimulation‐related complications documented by Cook and Braine et al. [24].

This study identified key variables affecting GCS score consistency through systematic screening of potential confounding factors and subsequent logistic regression analysis. We implemented strict inclusion and exclusion criteria to eliminate known confounders that interfere with GCS assessment, specifically sedative use, periorbital edema, and limb movement disorders. This methodological rigor aligns with confounders identified by Barami [22] and supports Reith et al.′s recommendation [15] for standardized assessment protocols, enhancing the interpretability of GCS reliability data.

Results demonstrated no significant correlation between GCS score consistency and nurses′ experience level, educational background, or professional grade (*p* > 0.05). This finding aligns with Reith et al.′s systematic review [15], which reported that while early studies suggested experience might affect GCS reliability, recent high‐quality research demonstrates minimal impact of professional background and experience. Our data further indicate that standardized training effectively mitigates these potential influences. These results have significant clinical implications: With standardized training, the GCS can be implemented reliably across healthcare professionals regardless of experience level or educational background.

Our analysis revealed no significant effect (*p* > 0.05) of visit timing (day vs. night shift) or transport mode (ambulance vs. nonambulance) on GCS score reliability. These results contrast with Kirschen et al. [26], who documented decreased consistency in eye and motor response scoring during night shifts, attributing this to insufficient stimulation or inadequate arousal of sleeping patients. Our study focused on emergency patients with assessments conducted upon arrival when patients typically demonstrate heightened alertness, thereby minimizing circadian rhythm effects. Additionally, standardized training protocols likely ensured methodological consistency across both day and night shifts. Our data indicate that properly standardized GCS assessment protocols maintain high reliability regardless of assessment timing or patient transport modality.

Patient clinical characteristics demonstrated significantly greater impact on GCS score reliability. Both ISS ≥ 16 (OR = 0.34, 95% CI: 0.24–0.47, *p* < 0.001) and traumatic brain injury (OR = 0.22, 95% CI: 0.16–0.31, *p* < 0.001) significantly reduced GCS score consistency. Notable GCS score variability was observed even between assessments conducted within brief intervals (≤ 5 min) for these patients. This finding reflects the inherent neurological instability and complexity in critically ill patients with traumatic brain injuries. Multiple factors may affect consciousness levels in traumatic brain injury patients, including secondary metabolic disorders, electrolyte abnormalities, medication effects, and seizures [27]. These confounding variables occur more frequently in high‐ISS patients and potentially contribute to increased GCS score volatility. Additionally, neurological status in traumatic brain injury patients can fluctuate rapidly, particularly during the acute phase, compounding assessment complexity. The GCS’s role as a “key clinical indicator of traumatic brain injury and overall injury severity” is particularly relevant, as our findings indicate that patients requiring the most precise GCS assessment paradoxically present the greatest assessment challenges.

This study yields significant clinical and educational implications within China’s developing prehospital triage infrastructure. Our findings confirm that properly trained nurses can reliably utilize GCS regardless of experience level—critical for ensuring teamwork and care continuity in acute settings. The identification of ISS and traumatic brain injury as key reliability factors necessitates heightened vigilance when assessing these high‐risk patients, potentially requiring more frequent neurological status evaluations.

Additionally, our findings validate the “sub‐score first” approach to GCS assessment. In emergency trauma settings, clinicians should emphasize individual GCS component scores rather than composite totals to prevent triage misclassification and treatment delays stemming from total score limitations. This approach enhances GCS clinical utility while providing a reliable framework for granular neurological recovery monitoring.

Our results establish an empirical foundation for enhancing China’s trauma care system. China’s limited prehospital trauma triage mechanisms currently result in provincial trauma centers receiving excessive minor‐to‐moderate cases. Enhancing in‐hospital GCS assessment reliability, particularly in resource‐constrained environments, optimizes trauma triage, improves critical patient identification, and strengthens clinical decision‐making foundations. Standardized training programs represent a cost‐effective intervention that may alleviate emergency department overcrowding and optimize healthcare resource allocation.

Inter‐rater variability decreases significantly through structured assessment processes, standardized stimulus protocols, and uniform scoring criteria—maintaining assessment consistency even in high‐intensity acute care environments. These findings inform emergency nursing education protocols and justify incorporating standardized GCS training within continuing education curricula.

Future research should employ multicenter designs to enhance external validity and confirm applicability across diverse hospital settings and geographic regions. Given the significant impact of ISS and traumatic brain injury on GCS reliability, future studies should focus on optimizing assessment protocols for these specific populations, potentially necessitating development of refined neurological assessment instruments. Additionally, further research should examine standardized training longevity and optimal GCS integration within electronic health records to enhance documentation accuracy and clinical utility. Finally, future research should investigate relationships between GCS scores and specific clinical decisions (trauma team activation, CT scanning, surgical interventions), validate standardized GCS assessment impact on patient outcomes, and establish comprehensive evidence for advancing China’s trauma care system.

### 5.1. Limitations

This study has several notable limitations. First, its single‐center design potentially limits external validity, particularly considering the regional variations in medical resources and trauma care quality across China. Second, despite excluding known confounding factors that influence GCS scores, unidentified variables inherent to clinical settings may restrict the generalizability of results to practical applications. Third, assessments conducted in actual clinical environments were susceptible to emergency situations and workplace stress—factors more easily controlled in experimental studies. While this approach enhanced ecological validity, it likely introduced additional variability, especially in high‐intensity emergency medical service (EMS) settings. Finally, although our overall sample size was substantial (*n* = 875), certain subgroup analyses were underpowered, potentially affecting statistical validity. Specifically, insufficient sample sizes for patients with particular traumatic brain injury types or extremely severe trauma (indicated by very high ISS) limited our ability to determine GCS score reliability in these specialized populations.

Despite these limitations, the demonstrated effectiveness of our standardized GCS training protocol provides strong evidence for its transferability to other similar healthcare settings. The systematic nature of our 19‐h training curriculum, the clear competency verification standards, and the resulting high inter‐rater reliability metrics (κw: 0.85–0.96; ICC: 0.85–0.96) establish a replicable framework that can be adapted across different institutions. Importantly, our finding that nurse experience, education level, and professional grade did not significantly influence GCS scoring consistency following standardized training suggests that this intervention can be effectively implemented regardless of staff demographics, making it particularly valuable for healthcare systems with diverse nursing backgrounds. The standardized assessment protocols, including structured pain stimulation techniques and electronic documentation systems, represent easily adoptable practices that address the core methodological challenges identified in previous GCS reliability research.

## 6. Conclusion

After standardized training, emergency nurses demonstrated high inter‐rater reliability in GCS application, though consistency in motor response scores and total GCS scores remains suboptimal. Patient factors—particularly ISS values and traumatic brain injury presence—significantly influenced GCS score consistency, while nurses′ professional experience and education level showed no substantial effect on reliability. These findings underscore the necessity for standardized GCS assessment protocols, especially regarding pain stimulus application, and emphasize the importance of heightened vigilance when evaluating critically ill patients and those with traumatic brain injuries.

In China’s developing prehospital triage infrastructure, this research provides empirical evidence for enhancing neurological assessment quality in hospital trauma cases, potentially improving triage decisions and treatment planning. Implementing standardized GCS assessment protocols represents an effective strategy for accurate identification of critically injured patients and optimal resource allocation in high‐intensity clinical environments. Future practice should adopt an “item‐first” assessment approach that values individual component responses over total scores, thereby improving neurological assessment accuracy and clinical decision quality. To facilitate this approach, clinical information systems should be designed to prominently display individual GCS components (eye, verbal, and motor responses) alongside total scores in patient dashboards and documentation interfaces, enabling clinicians to readily access the detailed neurological information necessary for optimal clinical decision‐making. This strategy will enhance emergency care standards while establishing a foundation for advancing China’s trauma care system.

## Disclosure

All authors read, provided comments, and approved the final manuscript.

## Conflicts of Interest

The authors declare no conflicts of interest.

## Author Contributions

Y.Z. and C.L. contributed equally to this work. Y.Z., C.L., and X.J. involved in drafting the manuscript or revising it critically for important intellectual content. Y.Z. and C.L. made substantial contributions to conception and design, acquisition of data, and analysis and interpretation of data. Y.Z. and C.L. have contributed equally to this work.

## Funding

Funding for this research was provided by Jiangsu Province Capability Improvement Project through Science, Technology and Education‐Jiangsu Provincial Medical Key Discipline (grant number: ZDXK202213), 2020 Provincial Financial Support for Clinical Key Specialty Project [Su Finance (2020) No. 155], and 2021 Specialist Capacity Building Project [Su Finance (2021) No. 79].

## Data Availability

The datasets used in the present study are available from the first author and corresponding authors on reasonable request.

## References

[1] Siti N. , Kamal A. F. , Dewi I. , Mansyur M. , and Bardosono S. , Trauma Team Activation in the Emergency Department: A Literature Review of Criteria, Processes and Outcomes, MJMHS. (2024) 20, no. 1, 323–329, 10.47836/mjmhs.20.1.40.

[2] Teasdale G. and Jennett B. , Assessment of Coma and Impaired Consciousness. A Practical Scale, Lancet (London, England). (1974) 2, no. 7872, 81–84, 10.1016/s0140-6736(74)91639-0, 2-s2.0-0016391336. 4136544

[3] Bieler D. , Trentzsch H. , Franke A et al., Evaluation of a Standardized Instrument for Post Hoc Analysis of trauma-team-activation-criteria in 75,613 Injured Patients an Analysis of the TraumaRegister DGU®, European Journal of Trauma and Emergency Surgery. (2022) 48, no. 2, 1101–1109, 10.1007/s00068-021-01668-2. 33876258 PMC9001544

[4] DiBrito S. R. , Cerullo M. , Goldstein S. D. , Ziegfeld S. , Stewart D. , and Nasr I. W. , Reliability of Glasgow Coma Score in Pediatric Trauma Patients, Journal of Pediatric Surgery. (2018) 53, no. 9, 1789–1794, 10.1016/j.jpedsurg.2017.12.027, 2-s2.0-85041699486. 29429772

[5] Dehli T. , Monsen S. A. , Fredriksen K. , and Bartnes K. , Evaluation of a Trauma Team Activation Protocol Revision: A Prospective Cohort Study, Scandinavian Journal of Trauma, Resuscitation and Emergency Medicine. (2016) 24, no. 1, 10.1186/s13049-016-0295-3, 2-s2.0-84983651736. PMC5000402 27561336

[6] Liu T. and Bai X. J. , Trauma Care System in China, Chinese Journal of Traumatology. (2018) 21, no. 2, 80–83, 10.1016/j.cjtee.2017.06.004, 2-s2.0-85037724611. 29246656 PMC5911711

[7] Teasdale G. , Maas A. , Lecky F. , Manley G. , Stocchetti N. , and Murray G. , The Glasgow Coma Scale at 40 years: Standing the Test of Time, The Lancet Neurology. (2014) 13, no. 8, 844–854, 10.1016/S1474-4422(14)70120-6, 2-s2.0-84904270315. 25030516

[8] Kliem P. S. C. , Tisljar K. , Grzonka P et al., Effects of a Scoring Aid on Glasgow Coma Score Assessment and Physicians′ Comprehension: A Simulator-Based Randomized Clinical Trial, Journal of Neurology. (2024) 272, no. 1, 10.1007/s00415-024-12825-z. PMC11638317 39666141

[9] Kohn M. A. , Hammel J. M. , Bretz S. W. , and Stangby A. , Trauma Team Activation Criteria as Predictors of Patient Disposition from the Emergency Department, Academic Emergency Medicine. (2004) 11, no. 1, 1–9, 10.1197/j.aem.2003.08.011, 2-s2.0-0346094145. 14709422

[10] Mehta R. and Chinthapalli K. , Glasgow Coma Scale Explained, BMJ. (2019) 365, 10.1136/bmj.l1296, 2-s2.0-85065327110. 31048343

[11] Momenyan S. , Mousavi S. M. , Dadkhahtehrani T et al., Predictive Validity and Inter-Rater Reliability of the Persian Version of Full Outline of Unresponsiveness Among Unconscious Patients with Traumatic Brain Injury in an Intensive Care Unit, Neurocritical Care. (2017) 27, no. 2, 229–236, 10.1007/s12028-016-0324-0, 2-s2.0-85008195222. 28054286

[12] Gill M. R. , Reiley D. G. , and Green S. M. , Interrater Reliability of Glasgow Coma Scale Scores in the Emergency Department, Annals of Emergency Medicine. (2004) 43, no. 2, 215–223, 10.1016/S0196-0644(03)00814-X, 2-s2.0-0942301425. 14747811

[13] Fischer M. , Rüegg S. , Czaplinski A et al., Inter-Rater Reliability of the Full Outline of Unresponsiveness Score and the Glasgow Coma Scale in Critically Ill Patients: A Prospective Observational Study, Critical Care. (2010) 14, no. 2, 10.1186/cc8963, 2-s2.0-77950663443. PMC2887186 20398274

[14] Reith F. C. M. , Van den Brande R. , Synnot A. , Gruen R. , and Maas A. I. R. , The Reliability of the Glasgow Coma Scale: A Systematic Review, Intensive Care Medicine. (2016) 42, no. 1, 3–15, 10.1007/s00134-015-4124-3, 2-s2.0-84948576242. 26564211

[15] Reith F. C. , Synnot A. , Van Den Brande R. , Gruen R. L. , and Maas A. I. , Factors Influencing the Reliability of the Glasgow Coma Scale: A Systematic Review, Neurosurgery. (2017) 80, no. 6, 829–839, 10.1093/neuros/nyw178, 2-s2.0-85015728060. 28327922

[16] Vink P. , Tulek Z. , Gillis K et al., Consciousness Assessment: A Questionnaire of Current Neuroscience Nursing Practice in Europe, Journal of Clinical Nursing. (2018) 27, no. 21-22, 3913–3919, 10.1111/jocn.14614, 2-s2.0-85052641153. 29989228

[17] Reith F. C. M. , Brennan P. M. , Maas A. I. R. , and Teasdale G. M. , Lack of Standardization in the Use of the Glasgow Coma Scale: Results of International Surveys, Journal of Neurotrauma. (2016) 33, no. 1, 89–94, 10.1089/neu.2014.3843, 2-s2.0-84952942148. 25951090

[18] Brennan P. M. , Whittingham C. , Sinha V. D. , and Teasdale G. , Assessment of Level of Consciousness Using Glasgow Coma Scale Tools, BMJ. (2024) 384, 10.1136/bmj-2023-077538. 38278534

[19] Gao Y. , Liao L. P. , Chen P et al., Application Effect for a Care Bundle in Optimizing Nursing of Patients with Severe Craniocerebral Injury, World Journal of Clinical Cases. (2021) 9, no. 36, 11265–11275, 10.12998/wjcc.v9.i36.11265. 35071557 PMC8717492

[20] Healey C. , Osler T. M. , Rogers F. B et al., Improving the Glasgow Coma Scale Score: Motor Score Alone is a Better Predictor, The Journal of Trauma, Injury, Infection, and Critical Care. (2003) 54, no. 4, 671–680, 10.1097/01.Ta.0000058130.30490.5d, 2-s2.0-0038747142. 12707528

[21] Kupas D. F. , Melnychuk E. M. , and Young A. J. , Glasgow Coma Scale Motor Component (“Patient Does Not Follow Commands”) Performs Similarly to Total Glasgow Coma Scale in Predicting Severe Injury in Trauma Patients, Annals of Emergency Medicine. (2016) 68, no. 6, 744–50.e3, 10.1016/j.annemergmed.2016.06.017, 2-s2.0-84998694307. 27436703

[22] Barami K. , Confounding Factors Impacting the Glasgow Coma Score: A Literature Review, Neurological Research. (2024) 46, no. 5, 479–486, 10.1080/01616412.2024.2329860. 38497232

[23] Kebapçı A. , Dikeç G. , and Topçu S. , Interobserver Reliability of Glasgow Coma Scale Scores for Intensive Care Unit Patients, Critical Care Nurse. (2020) 40, no. 4, e18–e26, 10.4037/ccn2020200. 32737493

[24] Cook N. F. , Braine M. E. , and Trout R. , Nurses′ Understanding and Experience of Applying Painful Stimuli when Assessing Components of the Glasgow Coma Scale, Journal of Clinical Nursing. (2019) 28, no. 21-22, 3827–3839, 10.1111/jocn.15011, 2-s2.0-85071836087. 31343105

[25] Basauhra Singh H. K. , Chong M. C. , Thambinayagam H. C et al., Assessing Nurses Knowledge of Glasgow Coma Scale in Emergency and Outpatient Department, Nursing Research and Practice. (2016) 2016, 1–5, 10.1155/2016/8056350. PMC5156803 28044104

[26] Kirschen M. P. , Snyder M. , Smith K et al., Inter-Rater Reliability Between Critical Care Nurses Performing a Pediatric Modification to the Glasgow Coma Scale, Pediatric Critical Care Medicine. (2019) 20, no. 7, 660–666, 10.1097/PCC.0000000000001938, 2-s2.0-85069271918. 30946292

[27] Maas A. I. R. , Menon D. K. , Manley G. T et al., Traumatic Brain Injury: Progress and Challenges in Prevention, Clinical Care, and Research, The Lancet Neurology. (2022) 21, no. 11, 1004–1060, 10.1016/s1474-4422(22)00309-x. 36183712 PMC10427240

